# Supplementary material for: Diagnostic accuracy of contemporary and high-sensitivity cardiac troponin assays used in serial testing, versus single-sample testing as a comparator, to triage patients suspected of acute non-ST-segment elevation myocardial infarction: a systematic review protocol
Source: BMJ Open. 2019 Mar 30;9(3):e026012. doi: 10.1136/bmjopen-2018-026012 (PMC6475186; doi:10.1136/bmjopen-2018-026012)
Supplement: Supplementary file 1 [file bmjopen-2018-026012supp001.pdf]

SUPPLEMENTARY FILE

Appendix 1 Search strategy for Embase

----

1 exp Chest Pain/

2 (chest adj2 pain*).ti,ab.

3 chest discomfort.ti,ab.

4 exp Acute Coronary Syndrome/

5 acute coronary syndrome*.ti,ab.

6 ACS.ti,ab.

7 exp Angina, Unstable/

8 unstable angina.ti,ab.

9 Myocardial Infarction/di

10 myocardial infarction.ti,ab.

11 heart attack.ti,ab.

12 or/1-11

13 cardiac troponin*.ti,ab.

14 high* sensitiv* troponin*.ti,ab.

15 hs-ctn*.ti,ab.

16 elecsys.ti,ab.

17 architectstat.ti,ab.

18 architect stat.ti,ab.

19 accutni*.ti,ab.

20 accutnl*.ti,ab.

21 centaur ultra.ti,ab.

22 HISCL.ti,ab.

23 vidas.ti,ab.

24 lumipulse.ti,ab.

25 serial troponin.ti,ab.

26 contemporary troponin.ti,ab.

27 heart adp.ti,ab.

28 (EDACS and troponin).ti,ab.

29 (Adapt adp and troponin).ti,ab.

30 (GRACE and troponin).ti,ab.

31 (TIMI and troponin).ti,ab.

32 or/13-31

33 exp Emergency Service, Hospital/

34 (emergency adj (room* or department*)).ti,ab.

35 (ER or ED).ti,ab.

36 (presenting or presented).ti,ab.

37 presentation.ti,ab.

38 (admission or admitted).ti,ab.

39 chest pain unit.ti,ab.

40 or/33-39

41 12 and 32 and 40

42 limit 41 to yr="2006 -Current"

Appendix 2 Data extraction form

General information:

Author

Title

Journal (incl. volume and page)

Year of publication

Institution

Country

Language

Translation details (if applicable)

Funding

Ethical approval

Characteristics of the study:

Study design

- Prospective vs. retrospective
- Single-gate vs. two-gate
- Multi-test design vs. randomized design vs. non-comparative design (i.e., single test)
- Multi-centre vs. single centre

Inclusion criteria

Exclusion criteria

Description of clinical setting, referral and selection process

Study years

Number of participants:

Eligible

Enrolled

Completed index test

Completed reference standard

Analysed

Excluded/dropped out (plus reasons)

Participants' characteristics:

Age: mean (SD)/median (IQR), range

Sex (n/% of male patients enrolled)

Time from symptom onset to presentation: mean (SD)/median (IQR), range

Risk score (e.g., TIMI, HEART, NCPR, GRACE, and etc.)

Other relevant information (smoking status, hypertension, diabetes, dyslipidaemia, prior history of MI/AP)

Prior tests (symptoms, vital signs, ECG)

Index test:

Assay

Description of decision rule (if applicable)

Cut-off values for positive disease

Cut-off values for negative disease

Blinding to the reference standard

Blinding to other clinical information

Reference standard:

Assay used to make a diagnosis

Definition of MI

- Single vs. composite vs. any rule vs. expert consensus

Assessors

- Number (kappa if multiple assessors involve)
- Experience
- Blinding to the index test
- Blinding to other clinical information

Test accuracy data

Number of TPs, FPs, FNs, TNs

Reported sensitivity, specificity, PPV, NPV

ROC curve (if reported)

Appendix 3 Criteria for rating the methodological quality of the included studies based on the QUADAS-2 tool

1. Domain: Patient selection
   a. Risk of bias

Question 1: Was a consecutive or random sample of patients enrolled? 'Yes' if consecutive or random sampling explicitly stated and described in sufficient detail; 'No' if non-consecutive or convenience sampling was used, and 'Unclear' if no sufficient information was provided to make a decision.

Question 2: Did the study avoid inappropriate exclusions? 'Yes' if all patients suspected of ACS that would normally undergo cardiac troponin testing were included; 'No' if relevant patient groups (e.g. patients with no history of CAD) were excluded and 'Unclear' if the reported data did not allow to make a judgement.

   b. Concerns regarding applicability

Are there concerns that the included patients and setting do not match the review question? 'No' if unselected patients >18 years of age presenting to the ED with symptoms suggestive of non-ST-segment elevation myocardial infarction included; 'Yes' if the patients or the setting did not match the review question (e.g. patients with ST-segment elevation included); and 'Unclear' if unspecified.

2. Domain: Index test
   a. Risk of bias

Question 1: If a threshold was used, was it pre-specified? 'Yes' if the results for at least one pre-specified threshold were reported; 'No' if ROC-optimisation or other methods were used to define a threshold.

   b. Concerns regarding applicability

Are there concerns that the index test, its conduct, or its interpretation differ from the review question? 'No' if the assay was used according to the manufacturer's recommendations; 'Yes' otherwise.

3. Domain: Reference standard
   a. Risk of bias

Question 1: Is the reference standard likely to classify correctly the target condition? 'Yes' if the diagnosis of myocardial infarction was adjudicated independently by two qualified clinicians according to the current universal definition (Thygesen 2012) and the method included high-sensitivity troponin assay; 'No' if not (including studies using an earlier generation of troponin assay or other biomarker as a reference test for myocardial necrosis) and 'Unclear' if not reported in sufficient detail.

Question 2: Was the reference standard independent from the index test (i.e. the index test did not form part of the reference standard)? 'Yes' if different troponin assay was used; 'No' if the results from the index test were included in the reference standard; and 'Unclear' if not reported in sufficient detail.

Question 3: Were the results from the reference standard interpreted without knowledge of the results from the index test? 'Yes' or 'No' if explicitly stated, 'Unclear' if not reported.

b. Concerns regarding applicability

Are there concerns that the target condition as defined by the reference standard does not match the review question? ‘No’ if the target condition was Non-ST-segment Elevation Myocardial Infarction (NSTEMI), diagnosed according to the current universal definition; ‘Yes’ if patients with ST-segment elevation on the initial ECG were included or the target condition deviated in some other way from the above definition.

4. Domain: Flow and timing
   a. Risk of bias

Question 1: Was there an appropriate interval between the index test and reference standard? ‘Yes’ if the reference test was done within 24 hours of the index test; ‘No’ if more than 24 hours and ‘Unclear’ if not reported.

Question 2: Did all patients receive a reference standard? ‘Yes’ if all patients included in the analysis received a reference standard; ‘No’ if some patients were included in the analysis without receiving a reference standard; ‘Unclear’ otherwise.

Question 3: Did all patients receive the same reference standard? ‘Yes’ if all patients received the same reference standard; ‘No’ otherwise and ‘Unclear’ if insufficient information.

Question 4: Were all patients included in the analysis? ‘Yes’ if all patients enrolled in the study were accounted for and included in the analysis; ‘No’ if patients who were enrolled in the study were excluded from the analysis, and ‘Unclear’ if insufficient data were available to decide.

Rules for producing an overall risk of bias rating for each domain:

• If all signalling questions within a domain are answered ‘Yes’ the overall domain rating is ‘Low risk of bias’

• If one or more signalling questions within a domain are answered ‘No’ the overall domain rating is ‘High risk of bias’

• If one or more signalling questions in the domain are answered ‘Unclear’ while the remaining signalling questions are answered ‘Yes’ then the overall domain rating is ‘Unclear risk of bias’.
