# Supplementary material for: Diagnostic accuracy of contemporary and high-sensitivity cardiac troponin assays used in serial testing, versus single-sample testing as a comparator, to triage patients suspected of acute non-ST-segment elevation myocardial infarction: a systematic review protocol
Source: BMJ Open. 2019 Mar 30;9(3):e026012. doi: 10.1136/bmjopen-2018-026012 (PMC6475186; doi:10.1136/bmjopen-2018-026012)
Supplement: Author's manuscript [file bmjopen-2018-026012.draft_revisions.pdf]

BMJ Open is committed to open peer review. As part of this commitment we make the peer review history of every article we publish publicly available.

When an article is published we post the peer reviewers' comments and the authors' responses online. We also post the versions of the paper that were used during peer review. These are the versions that the peer review comments apply to.

The versions of the paper that follow are the versions that were submitted during the peer review process. They are not the versions of record or the final published versions. They should not be cited or distributed as the published version of this manuscript.

BMJ Open is an open access journal and the full, final, typeset and author-corrected version of record of the manuscript is available on our site with no access controls, subscription charges or pay-per-view fees (http://bmjopen.bmj.com).

If you have any questions on BMJ Open's open peer review process please email editorial.bmjopen@bmj.com

BMJ Open

Diagnostic accuracy of sensitive and high-sensitivity cardiac troponin assays used in serial testing to triage patients suspected of acute myocardial infarction: A systematic review protocol

Journal:	*BMJ Open*
Manuscript ID	bmjopen-2018-026012
Article Type:	Protocol
Date Submitted by the Author:	15-Aug-2018

Complete List of Authors:	Zhelev, Zhivko; University of Exeter, Exeter Test Group, University of Exeter Medical School<br>Ohtake, Hirotaka; Fujita Health University School of Medicine, Department of Emergency and General Internal Medicine<br>Iwata, Mitsunaga; Fujita Health University School of Medicine, Department of Emergency and General Internal Medicine<br>Terasawa, Teruhiko ; 1Fujita Health University School of Medicine, Department of Emergency and General Internal Medicine<br>Rogers, Morwenna; University of Exeter Medical School, NIHR CLAHRC South West Peninsula<br>Peters, Jaime; University of Exeter, Exeter Test Group, University of Exeter Medical School<br>Hyde, Chris; University of Exeter, Exeter Test Group, University of Exeter Medical School

Keywords:	acute coronary syndrome, Myocardial infarction < CARDIOLOGY, troponin, emergency department, meta-analysis, sesnsitivity and specificity

SCHOLARONE™
Manuscripts

Title: Diagnostic accuracy of sensitive and high-sensitivity cardiac troponin assays used in serial testing to triage patients suspected of acute myocardial infarction: A systematic review protocol

Corresponding author:

Full name: Zhivko Zhelev

Postal address: Exeter Test Group, PenCLAHRC
University of Exeter Medical School
South Cloisters (Room 3.09)
St Luke's Campus
EXETER EX1 2LU

E-mail: Z.Zhelev@exeter.ac.uk

Tel: 0044 (0)1392 726093

Co-authors:

Hirotaka Ohtake, MD; Department of Emergency and General Internal Medicine, Fujita Health University School of Medicine, Toyoake, Japan

Mitsunaga Iwata, MD, PhD; Department of Emergency and General Internal Medicine, Fujita Health University School of Medicine, Toyoake, Japan

Teruhiko Terasawa MD PhD, Department of Emergency and General Internal Medicine, Fujita Health University School of Medicine, Toyoake, Japan

Morwenna Rogers, PenCLAHRC, University of Exeter Medical School, University of Exeter, Exeter, UK

For peer review only

For peer review only - http://bmjopen.bmj.com/site/about/guidelines.xhtml

Jaime Peters PhD, Exeter Test Group, University of Exeter Medical School, University of Exeter, Exeter, UK

Prof Christopher Hyde, MD PhD, Exeter Test Group, University of Exeter Medical School, University of Exeter, Exeter, UK

Word count: 3612

For peer review only

For peer review only - http://bmjopen.bmj.com/site/about/guidelines.xhtml

ABSTRACT

Introduction

Although the new generation of cardiac troponin assays have revolutionised the triage and diagnosis of myocardial infarction (MI), their application in the triage of patients with suspected acute coronary syndrome (ACS) requires further investigation. The objectives of the current systematic review are to evaluate the diagnostic accuracy of sensitive and high-sensitivity cardiac troponin assays when used in serial testing to identify patients with MI in the emergency department (ED).

Methods and analysis

We will conduct systematic searches of MEDLINE, EMBASE, Science Citation Index, the Cochrane Database of Systematic Reviews and the CENTRAL database, and hand searches to identify additional titles or unpublished data. No restrictions on language or publication status will be applied. Two review authors will independently screen studies for inclusion, extract data from eligible studies and assess their methodological quality using QUADAS-2. Studies will be included if they evaluate the accuracy of sensitive or high-sensitivity cardiac troponin assays used in serial testing in patients presenting to the ED with suspicion of MI. Estimates of sensitivity and specificity from each study will be presented in forest plots and in the receiver operating characteristics (ROC) space. If appropriate, we will pool the results using Bayesian hierarchical models that allow correction for imperfect reference standard. We will obtain summary estimates of sensitivity and specificity of alternative testing protocols and compare their accuracy. We will also investigate the impact of pre-specified sources of heterogeneity and methodological quality items. If pooling of results is

For peer review only - http://bmjopen.bmj.com/site/about/guidelines.xhtml

considered inappropriate, we will present our findings in tables and diagrams and will describe them narratively.

Ethics and dissemination

No formal ethical approval will be sought, but we will report on the ethical approval of the included studies. Dissemination of findings will be through publications in peer-reviewed journals, presentations at conferences and the web-sites of the universities.

PROSPERO registration number: CRD42018106379

Keywords: acute coronary syndrome, myocardial infarction, troponin, emergency department, systematic review, meta-analysis, sensitivity and specificity

ARTICLE SUMMARY

Strengths and limitations of this study

- Systematic and comprehensive searches to identify all published and unpublished studies
- Robust procedures for screening, data extraction and methodological quality assessment, based on pre-specified criteria
- Statistical methods for pooling results that allow correction for an imperfect reference standard
- If the number of studies is too small and heterogeneity in study-level estimates is considered too great, we may not be able to obtain summary estimates of sensitivity and specificity.

For peer review only - http://bmjopen.bmj.com/site/about/guidelines.xhtml

INTRODUCTION

Coronary heart disease is one of the most common chronic conditions causing more than 7 million deaths worldwide each year [1]. The underlying pathogenesis is progressive accumulation of atheromatous plaque on the walls of the coronary arteries which, in its advanced stages, obstructs the normal blood flow and causes myocardial ischemia. If the plaque gets ruptured, clots may form and limit, or completely cut off, the blood supply to part of the heart muscle. This life-threatening condition is known as 'acute coronary syndrome' and presents with chest pain, dyspnoea, nausea and other unspecific symptoms. It includes type 1 myocardial infarction (MI), if there is evidence of permanent damage to the heart muscle, or unstable angina, if no evidence of myocardial necrosis is detected [2]. Myocardial infarction could also happen in the absence of acute coronary syndrome, as a result of other processes, including mismatch between myocardial oxygen supply and demand (type 2 MI) [3].

Patients with MI require urgent specialist treatment and their outcomes depend on a timely and accurate diagnosis. However, diagnosis in an emergency setting could be challenging as clinical symptoms lack sufficient sensitivity and specificity [4] and a normal electrocardiogram (ECG) does not rule out the condition. Therefore, in patients suspected of MI but with non-diagnostic ECG, the current guidelines recommend determination of troponin T or I, highly specific biomarkers of cardiac necrosis, both as a triage tool and in establishing the final diagnosis [5 ,6].

Although cardiac troponins are specific to myocardial necrosis, they are not specific to MI and elevated concentration could be found in a number of other acute and chronic conditions that cause myocardial injury. The main difference between acute MI and most of

For peer review only

the other conditions is the pattern of dynamic changes. Acute MI is characterised by a sharp increase in the bloodstream concentration of troponin in the first 24 hours of symptom onset, reflecting the progressive nature of the condition. Consequently, a significant difference in troponin values from two consecutive samples (usually referred to as 'delta troponin') is interpreted as an indication that the most likely underlying condition is acute MI. Another consideration is the fact that in the first two hours of symptom onset the level of cardiac troponin in the bloodstream may not exceed the 99th centile, which is an arbitrary cutoff used to dichotomise patients as having or not having myocardial injury. Therefore, a single determination early in the diagnostic process might miss patients with an evolving MI.

Sensitive and high-sensitivity cardiac troponin assays

Older generations of cardiac troponin assays lacked the precision and sensitivity to identify a small increase in the bloodstream level of the biomarker soon after onset of symptoms. Therefore, testing was done 6 – 12 hours after presentation to the ED, causing considerable delay in diagnosis and treatment, and unnecessary hospital admissions of patients without MI [7]. In the past ten years sensitive and high-sensitivity cardiac troponin assays have been developed and adopted in clinical practice. They can measure small concentrations of troponin with high levels of precision, which means that MI can be identified much earlier in the triage or ruled out in those with low pre-test probability.

Given the large number of cardiac troponin assays marketed as 'high-sensitivity', recent efforts have been made to define criteria that could inform policy and clinical decisions. In the development of their guidance on early rule out of acute MI, [5] the UK's National Institute for Health and Care Excellence (NICE) defined a high-sensitivity cardiac troponin assay as one with coefficient of variation (CV) ≤10% at the 99th centile and the ability to

For peer review only - http://bmjopen.bmj.com/site/about/guidelines.xhtml

measure concentrations in at least 50% of the reference population. The systematic review that informed the guidance identified only three commercially available assays that met the above criteria: the Elecsys Troponin T high-sensitive assay (Roche Diagnostics), the ARCHITECTSTAT high sensitive troponin I (Abbott Laboratories) and the AccuTnI+3 (Beckman Coulter) [7]. The above definition is also incorporated in the European Society of Cardiology 2015 Guidelines for the management of acute coronary syndrome in patients without persistent ST-segment elevation. In this document, a distinction is made between 'sensitive' assays (able to detect cardiac troponin in approximately 20% – 50% of healthy individuals) and 'high-sensitivity' assays (approx. 50% - 90%).

Serial testing

Serial measurement of cardiac troponin is employed to increase both sensitivity and specificity of the diagnostic strategy. This usually involves one sample taken at presentation to the ED and another sample taken one or more hours later. The results from these two determinations and their difference ('delta troponin') are combined in a diagnostic rule to determine the likelihood of the symptoms being caused by an acute MI. The strategy takes advantage of the fact that in the first 24 hours of the onset of symptoms the level of cardiac troponin in the bloodstream is progressively rising. According to the current universal definition, diagnosis of MI requires that the patient's presentation is consistent with cardiac ischaemia; cardiac troponin values are above the 99th centile of the reference population and two consecutive determinations a few hours apart show significant change (e.g. >20%) in cardiac troponin level [8]. Such patients require urgent reperfusion to restore blood flow to the affected part of the heart and should immediately be admitted to hospital.

Clinical pathway

For peer review only - http://bmjopen.bmj.com/site/about/guidelines.xhtml

The generic clinical pathway is presented graphically in Figure 1. Most patients with symptoms suggestive of acute coronary syndrome are transported by ambulance or present directly to the ED [9]. Their initial triage includes clinical assessment, medical history and a 12-lead ECG. Patients with ST-segment elevation MI (STEMI) or other high-risk features are directly admitted to hospital. Low risk patients undergo troponin testing, with the first sample taken at presentation and the second sample one or more hours later. If both determinations are below the 99th centile and there is no evidence of evolving MI (e.g. rising troponin, new angina or other symptoms), the patient is diagnosed with unstable angina or alternative explanation of the symptoms is sought.

Diagnostic strategies used in the triage of patients suspected of MI

Taking advantage of the high sensitivity and precision of the new generation cardiac troponin assays, a range of diagnostic protocols addressing specific clinical scenarios have been devised. They could be summarised as follows:

- Single-sample rule-out: If the level of troponin in the first sample, taken two or more hours after the onset of symptoms, is well below the 99th centile (e.g. below the limit of detection), the probability of MI is also very low and the patient could be discharged or alternative explanation of the symptoms could be sought. For instance, a meta-analysis conducted by Chapman and colleagues [10] showed that the negative predictive value of Abbott ARCHITECT$_{STAT}$ high-sensitive cardiac troponin I assay (Abbott Laboratories) is 99.5%, when the cutoff is “< 5 ng/L”; the 99th centile of this assay is much higher: 34 ng/L for men and 16 ng/L for women [11]. Alternative early rule-out strategies combine a single sample high-sensitivity troponin with copeptin [12] or information from the ECG and the Thrombolysis in

For peer review only - http://bmjopen.bmj.com/site/about/guidelines.xhtml

Myocardial Infarction (TIMI) risk score [13 ,14]. This increases the negative predictive value of the protocol and the proportion of patients assigned to the rule-out group.

- Two-sample rule-out:
    - If the results from two samples taken one or more hours apart are below the 99th centile and there is no significant change, the probability of MI is low.
    - If both values are slightly above the 99th centile, but there is no change, the underlying cause might be a chronic condition, such as heart failure or myocarditis.
- Single-sample rule-in: In the context of myocardial ischemia, if the value from the first sample is well above the 99th centile, the probability of MI is high and the patient will most likely be admitted to hospital for further testing and treatment.
- Two-sample rule-in: The universal diagnosis of MI requires that at least one of two consecutive samples taken three or more hours apart produces a value above the 99th centile and there is a significant change in the concentration of cardiac troponin between the first and the second sample [8].

The above protocols could be combined in complex diagnostic strategies that use different positivity criteria to rule in, rule out or assign patients to an observational zone when. Such strategies enable clinicians to stratify patients early in the triage, so that appropriate referral and treatment decisions could be made without delay.

Rationale for conducting the review

Recent meta-analyses have focused on single-sample rule out protocols [10 ,15 ,16]. Such protocols have important implications for clinical practice, especially in terms of cost-effectiveness and quality of patient care: they could free up capacity required for patients

For peer review only - http://bmjopen.bmj.com/site/about/guidelines.xhtml

who need emergency treatment; reduce the number of unnecessary tests and the associated risks; the focus of clinical investigation could shift earlier to an alternative explanation of the patient's symptoms and, if appropriate, the patient could be discharged directly from the ED [9].

However, single-sample rule-out protocols are limited in their application, as many patients without MI will present with troponin values above the positivity threshold. Such patients will undergo a second troponin determination which, if the inter-sample time is three or more hours, as recommended in the current NICE guidance,[5] will result in the patients spending many hours in hospital. In some cases they will have to stay overnight, until a qualified clinician is available to review results and make a decision for transfer or discharge.

Various serial testing protocols have been devised and validated, including those with different cutoffs for rule-in, rule-out and shorter (one or two hours) inter-sample time. Such protocols could enable discharge or referral decisions to be made for a larger proportion of patients while still in the ED. A brief scoping of the literature showed considerable variation in these protocols and their accuracy, and we can assume that the variation in clinical practice is even greater. The differences concern:

- The analytical and diagnostic performance of different assays
- The positivity thresholds used to rule in and rule out
- The method of calculating delta troponin: absolute versus relative change (e.g. >4.5 ng/L vs. >20%), and
- The inter-sample time (1, 2 or more hours).

Other factors, such as the time from onset of symptoms, patient characteristics (age, sex, risk factors) and prior tests (e.g. ECG findings) are also likely to affect the performance of

For peer review only - http://bmjopen.bmj.com/site/about/guidelines.xhtml

For peer review only

the assays and contribute to the observed heterogeneity in study-level estimates [10]. This could be confusing when making decisions about which protocol to implement in practice, in order to achieve the best balance between accuracy and efficiency (the proportion of patients for whom a decision for discharge or admission to a specialist unit could be made early in the triage).

Therefore, we are proposing to systematically identify, review and summarise research evidence pertaining to the diagnostic accuracy of sensitive and high-sensitivity cardiac troponin assays, when used in serial testing to identify MI in symptomatic patients presenting to the ED. Our primary objective will be to obtain summary estimates of the accuracy of specific protocols and the proportion of patients assigned to an observational zone when different rule-in and rule-out criteria are used. Our secondary objectives will be to determine the relative accuracy of alternative protocols, including a direct comparison of single-sample and two-sample protocols; and to investigate the impact of protocol- and study-level characteristics on test accuracy estimates.

METHODS AND ANALYSIS

In preparing the protocol, we used the PRISMA-P reporting guidelines [17].

Eligibility criteria

Study design

We will include all primary diagnostic accuracy studies with single-gate design (also known as diagnostic cohort studies). Studies with two-gate design will be excluded due to inherent bias in the reported diagnostic accuracy estimates [18].

Participants

For peer review only - http://bmjopen.bmj.com/site/about/guidelines.xhtml

For peer review only

Only studies in patients 18 years of age or older, who present to the ED with suspicion of acute coronary syndrome, will be considered for inclusion. Studies in patients with STEMI will be excluded, but we will include studies analysing jointly STEMI and non-STEMI patients. We will include only data for non-STEMI, if such data could be obtained; otherwise, we will deal with this in the methodological quality assessment and analysis.

Index tests

The index tests are sensitive or high-sensitivity cardiac troponin assays used in serial measurement (two or more determinations) to identify myocardial infraction. To qualify as 'sensitive' or 'high-sensitivity' assays need to have CV ≤10% at the 99th percentile of normal; 'sensitive' assays should be able to detect cardiac troponin in 20% – 50% of healthy individuals and 'high-sensitivity' assays in 50% - 90%. We will include all cardiac troponin assays that meet the above criteria.

Target conditions and reference standard

Our primary target condition will be an index diagnosis of MI. Our secondary target conditions will be:

- A composite outcome of index MI and cardiac death within 30 days of the index presentation.
- A composite outcome of index MI, death and major adverse cardiac events (MACEs) including cardiac arrest, emergency revascularization procedure, cardiogenic shock, ventricular arrhythmia or high-degree atrioventricular block requiring intervention, if they occur within 30 days of the index presentation.

For peer review only - http://bmjopen.bmj.com/site/about/guidelines.xhtml

The reference standard is a final diagnosis of MI adjudicated by qualified clinicians according to the universal definition of MI [8]. The quality of the reference standard, such as independence from the index test, adjudication and blinding to the index test results, will be considered in the methodological quality assessment.

Outcomes

The main outcomes are test accuracy data reported as true positives, false positives, false negatives and true negatives for protocols with dichotomous outcomes, or three-by-two tables for protocols that use separate rule-in, rule-out and observational zone criteria. If the contingency table is not reported in the paper, we will derive data from the sensitivity and specificity estimates, the total number of patients and the proportion of those with the target condition. Studies that report only test accuracy estimates but insufficient detail to reconstruct the original contingency table will be included in the review, but will be excluded from the quantitative analysis. Studies that meet the rest of the criteria, but do not report test accuracy data will be excluded only after we have tried but failed to obtain the missing data.

Publication status and language

We will include conference abstracts and unpublished studies if they meet our eligibility criteria and we are able to obtain the necessary data. We will include all relevant papers regardless of the language in which they are published. If necessary, we will arrange translation or data extraction by a person fluent in the respective language.

Searches

For peer review only - http://bmjopen.bmj.com/site/about/guidelines.xhtml

We plan to perform a systematic search of the following electronic databases: Ovid MEDLINE and MEDLINE in-process, Ovid Embase, Science Citation Index (SCI), the CENTRAL database and Cochrane Database of Systematic Reviews (CDSR). Related systematic reviews will be examined for additional studies for inclusion. Our search strategy was developed by an information specialist (MR) and combines terms for the target condition, index tests and setting. The search strategy for Embase is provided as Appendix 1 in the Supplemental file, and the search strategies for the other databases could be obtained from the authors upon request.

In addition, we will conduct forward and backward searches of all included papers and other relevant publications (e.g. previous systematic reviews), and will contact experts in the field to check for missed titles or unpublished data.

Selection of studies for inclusion

The results from the electronic searches will be imported into a reference management software and de-duplicated. Two review authors will conduct all screening independently with disagreements resolved through discussion or arbitration. First, we will screen all titles and abstracts; the full text of any potentially relevant publications will be retrieved and assessed for eligibility against our inclusion criteria. The same procedure will be repeated for titles identified in the additional searches.

Data extraction

We have developed and piloted a data extraction form available in the online supplement (Appendix 2 in the Supplementary file). Data will be extracted for the following topics: publication details, study characteristics, patients, index tests, reference standard,

For peer review only - http://bmjopen.bmj.com/site/about/guidelines.xhtml

outcomes and methodological quality items. Two review authors will extract data independently and all disagreements will be resolved through discussion or arbitration. Study authors will be contacted for missing data, with reminders sent two weeks after the initial request.

Methodological quality assessment

We will assess the methodological quality of the included studies using a tailored version of the Quality Assessment of Diagnostic Accuracy Studies version 2 (QUADAS-2) tool. The tool consists of four domains: Patient selection, Index test, Reference standard and Flow and timing. Each domain is assessed for risk of bias and rated as “High risk”, “Low risk” or “Unclear risk”. In addition, the first three domains are assessed for applicability concerns and rated using the same categories. The authors of the tool recommend that it is tailored for each systematic review by adding or removing signalling questions [19]. A tailored version of the tool with details of the signalling questions, operational definitions and the rules for combining the answers to produce a domain-level rating is included in the Supplementary file (Appendix 3).

Assessment of publication bias

Given the limited knowledge of publication bias in diagnostic accuracy studies and the controversies around its assessment, [20 ,21] we will not use statistical methods to detect the presence of publication bias. We acknowledge, however, that publication bias might be present and have bearing on our findings. We will try to mitigate this by conducting comprehensive searches of the published research, actively seek to identify unpublished data and note the possibility of publication bias when reporting our findings.

For peer review only - http://bmjopen.bmj.com/site/about/guidelines.xhtml

Statistical analysis and data synthesis

Contingency tables will be reconstructed from the data reported in the papers or by entering sensitivity, specificity, total number of patients and the proportion of those with the target condition in the test accuracy calculator of the Cochrane Collaboration's Review Manager 5.3 (The Nordic Cochrane Centre, The Cochrane Collaboration, 2014). Sensitivity and specificity estimates with 95% confidence intervals will be calculated and presented in forest plots and in the receiver operating characteristics (ROC) space. Data will be grouped by individual assays and protocols. We will conduct visual inspection of each plot, to determine the level and potential sources of heterogeneity, and to decide whether meta-analysis is appropriate.

If a sufficient number of studies per protocol are available and the level of clinical and statistical between-study heterogeneity is low, we will conduct a meta-analysis by dichotomising the results from studies reporting more than two outcomes, such as rule-in, rule-out and an observational zone. First, we will combine the rule-in and observational zone counts to obtain a two-by-two table for the rule-out part of the protocol. Then, we will combine the rule-out and the observational zone to obtain a two-by-two table for the rule-in part. These will be included in separate meta-analyses, to obtain summary sensitivity and specificity for the rule-out and rule-in part of the protocol.

Since both the index tests and the reference standard are cardiac troponin assays for the detection of myocardial necrosis, we assume the presence of incorporation bias and will pool the results using the model developed by Dendukuri and colleagues [22]. The model, an extension of the hierarchical summary receiver operating characteristic (HSROC) model, allows correction for imperfect reference standard assuming an underlying continuous

For peer review only - http://bmjopen.bmj.com/site/about/guidelines.xhtml

latent variable. The same methods will also be used to investigate the comparative accuracy of alternative assays and protocols, and the impact of protocol- and study-level characteristics. As a sensitivity analysis, we will also analyse the data using the HSROC model where incorporation bias is not accounted for.

If we decide that pooling of results would be inappropriate (for instance, because the number of studies is too small and heterogeneity too great), we will summarise the results in tables and graphs, and will provide a narrative summary of the observed differences.

Investigation of heterogeneity:

The protocol- and study-level-characteristics explored will include the following potential sources of heterogeneity:

- Definition of the target condition: type 1 MI vs. type 1 and type 2 MI
- Different inter-sample time
- Age (≤65 or >65 years) and sex
- Time since symptom onset (≤2 or >2 hours)
- Presence of myocardial ischemia on electrocardiogram, e.g. ST-segment changes or T-wave inversion
- History of ischemic heart disease.

Sensitivity analysis

If appropriate, sensitivity analysis will be conducted to explore the impact of the methodological quality of the studies on summary estimates.

ETHICS AND DISSEMINATION

For peer review only - http://bmjopen.bmj.com/site/about/guidelines.xhtml

As this is a systematic review, we are not planning to obtain a formal ethical approval. However, we will extract and report data on the ethical approval of the included studies. The findings from the review will be disseminated through publications in peer-reviewed journals, presentations at conferences and using the web sites and networks of the two universities. Changes and amendments to the protocol will be acknowledged in all presentations and publications.

AUTHOR CONTRIBUTIONS

TT originated the idea; TT and ZZ drafted the initial version of the protocol; MR developed the search strategy and wrote the section on search methods; CH, JP, HO and MI reviewed the protocol and suggested amendments. All authors read and approved the final version of the protocol. TT and ZZ are guarantors of the review.

COMPETING INTERESTS STATEMENT

None declared.

PATIENT AND PUBLIC INVOLVEMENT

No patients have been involved in the development of the protocol.

ACKNOWLEDGEMENTS

None.

FUNDING

This research was funded in part by Fujita Health University, Japan, the Ministry of Education, Culture, Sports, Science and Technology (MEXT), Japan (grant numbers: 18K08902 and 26460755), and the National Institute for Health Research (NIHR)

For peer review only

Collaboration for Leadership in Applied Health Research and Care South West Peninsula (NIHR CLAHRC South West Peninsula). The views expressed are those of the author(s) and not necessarily those of the Fujita Health University, the MEXT, the NHS, the NIHR or the Department of Health and Social Care. The funders have no role in the development of the protocol.

DATA SHARING

Dataset will be published as a supplementary file.

REFERENCES

1. World Health Organization. Cardiovascular diseases (CVDs): Key facts. 17 May 2017. http://www.who.int/news-room/fact-sheets/detail/cardiovascular-diseases-(cvds) (date accessed 6 Aug 2018).
2. Ibanez B, James S, Agewall S, et al. 2017 ESC Guidelines for the management of acute myocardial infarction in patients presenting with ST-segment elevation. The Task Force for the management of acute myocardial infarction in patients presenting with ST-segment elevation of the European Society of Cardiology (ESC). *European Heart Journal* 2018;**39**(2):119-77.
3. McCarthy CP, Vaduganathan M, Januzzi JL, et al. Type 2 myocardial infarction—diagnosis, prognosis, and treatment. *JAMA* 2018;**320**(5):433-34.
4. Bruyninckx R, Aertgeerts B, Bruyninckx P, et al. Signs and symptoms in diagnosing acute myocardial infarction and acute coronary syndrome: a diagnostic meta-analysis. *The British journal of general practice : the journal of the Royal College of General Practitioners* 2008;**58**(547):105-11.
5. The National Institute for Health and Care Excellence. Myocardial infarction (acute): Early rule out using high-sensitivity troponin tests (Elecsys Troponin T high-sensitive, ARCHITECT STAT High Sensitive Troponin-I and AccuTnI+3 assays): Diagnostics guidance [DG15]. 2014. https://www.nice.org.uk/guidance/dg15 (date accessed 6 Aug 2018).
6. Roffi M, Patrono C, Collet JP, et al. 2015 ESC Guidelines for the management of acute coronary syndromes in patients presenting without persistent ST-segment elevation: Task Force for the Management of Acute Coronary Syndromes in Patients Presenting without Persistent ST-Segment Elevation of the European Society of Cardiology (ESC). *Eur Heart J* 2016;**37**(3):267-315.
7. Westwood M, van Asselt T, Ramaekers B, et al. High-sensitivity troponin assays for the early rule-out or diagnosis of acute myocardial infarction in people with acute chest pain: a systematic review and cost-effectiveness analysis. *Health technology assessment (Winchester, England)* 2015;**19**(44):1-234.
8. Thygesen K, Alpert JS, Jaffe AS, et al. Third universal definition of myocardial infarction. *Circulation* 2012;**126**(16):2020-35.
9. Rachuba S, Salmon A, Zhelev Z, et al. Redesigning the diagnostic pathway for chest pain patients in emergency departments. *Health care management science* 2018;**21**(2):177-91.

For peer review only - http://bmjopen.bmj.com/site/about/guidelines.xhtml

10. Chapman AR, Lee KK, McAllister DA, et al. Association of High-Sensitivity Cardiac Troponin I Concentration With Cardiac Outcomes in Patients With Suspected Acute Coronary Syndrome. *Jama* 2017;**318**(19):1913-24.
11. Lee GR, Browne TCA, Guest B, et al. Transitioning high sensitivity cardiac troponin I (hs-cTnI) into routine diagnostic use: More than just a sensitivity issue. *Practical Laboratory Medicine* 2016;**4**:62-75.
12. Balmelli C, Meune C, Twerenbold R, et al. Comparison of the performances of cardiac troponins, including sensitive assays, and copeptin in the diagnostic of acute myocardial infarction and long-term prognosis between women and men. *American heart journal* 2013;**166**(1):30-7.
13. Cullen L, Mueller C, Parsonage WA, et al. Validation of high-sensitivity troponin I in a 2-hour diagnostic strategy to assess 30-day outcomes in emergency department patients with possible acute coronary syndrome. *Journal of the American College of Cardiology* 2013;**62**(14):1242-49.
14. Than M, Cullen L, Aldous S, et al. 2-Hour accelerated diagnostic protocol to assess patients with chest pain symptoms using contemporary troponins as the only biomarker: the ADAPT trial. *Journal of the American College of Cardiology* 2012;**59**(23):2091-8.
15. Pickering JW, Than MP, Cullen L, et al. Rapid rule-out of acute myocardial infarction with a single high-sensitivity cardiac troponin t measurement below the limit of detection: A collaborative meta-analysis. *Annals of internal medicine* 2017;**166**(10):715-24.
16. Zhelev Z, Hyde C, Youngman E, et al. Diagnostic accuracy of single baseline measurement of Elecsys Troponin T high-sensitive assay for diagnosis of acute myocardial infarction in emergency department: systematic review and meta-analysis. *BMJ : British Medical Journal* 2015;**350:h15**.
17. Moher D, Shamseer L, Clarke M, et al. Preferred reporting items for systematic review and meta-analysis protocols (PRISMA-P) 2015 statement. *Systematic reviews* 2015;**4**:1.
18. Rutjes AW, Reitsma JB, Vandenbroucke JP, et al. Case-control and two-gate designs in diagnostic accuracy studies. *Clinical chemistry* 2005;**51**(8):1335-41.
19. Whiting PF, Rutjes AW, Westwood ME, et al. QUADAS-2: a revised tool for the quality assessment of diagnostic accuracy studies. *Annals of internal medicine* 2011;**155**(8):529-36.
20. Deeks JJ, Macaskill P, Irwig L. The performance of tests of publication bias and other sample size effects in systematic reviews of diagnostic test accuracy was assessed. *Journal of clinical epidemiology* 2005;**58**(9):882-93.
21. Macaskill P GC, Deeks JJ, Harbord RM, Takwoingi Y. Chapter 10: Analysing and Presenting Results. In: Deeks JJ BP, Gatsonis C, ed. Cochrane Handbook for Systematic Reviews of Diagnostic Test Accuracy Version 10: The Cochrane Collaboration, 2010.
22. Dendukuri N, Schiller I, Joseph L, et al. Bayesian meta-analysis of the accuracy of a test for tuberculous pleuritis in the absence of a gold standard reference. *Biometrics* 2012;**68**(4):1285-93.

FIGURE LEGENDS

Figure 1 Clinical pathway

For peer review only - http://bmjopen.bmj.com/site/about/guidelines.xhtml

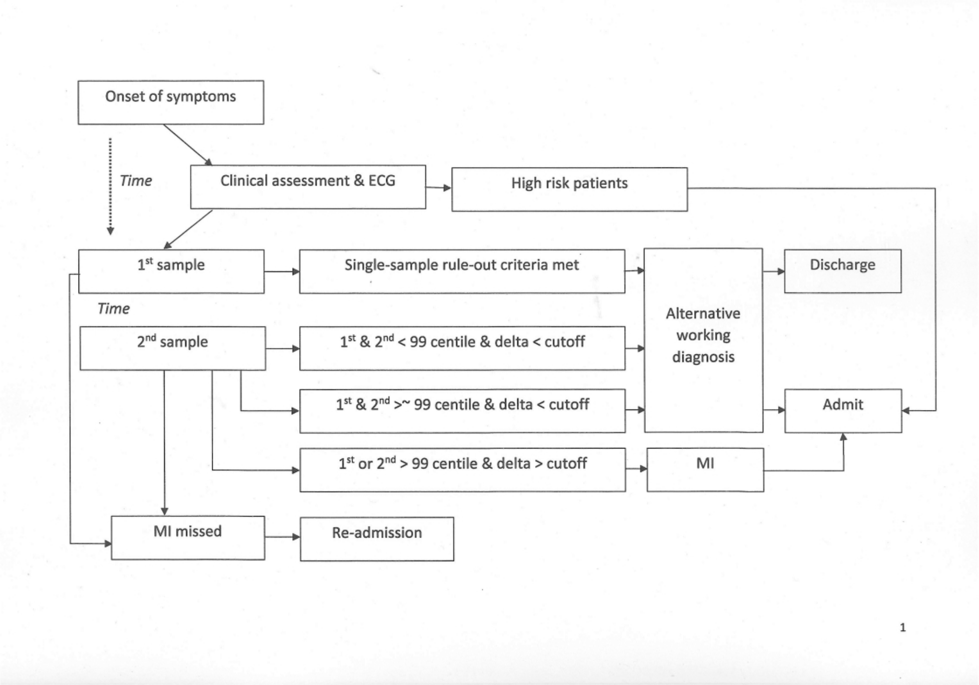

Figure 1 Clinical pathway

298x211mm (300 x 300 DPI)

SUPPLEMENTARY FILE

Appendix 1 Search strategy for Embase

----

1 exp Chest Pain/ (74337)

2 (chest adj2 pain*).ti,ab. (50081)

3 chest discomfort.ti,ab. (2099)

4 exp Acute Coronary Syndrome/ (47975)

5 acute coronary syndrome*.ti,ab. (45786)

6 ACS.ti,ab. (32678)

7 exp Angina, Unstable/ (21737)

8 unstable angina.ti,ab. (17861)

9 Myocardial Infarction/di (6151)

10 myocardial infarction.ti,ab. (235078)

11 heart attack.ti,ab. (5586)

12 or/1-11 (374553)

13 cardiac troponin*.ti,ab. (12673)

14 high* sensitiv* troponin*.ti,ab. (2434)

15 hs-ctn*.ti,ab. (1480)

16 elecsys.ti,ab. (1808)

17 architectstat.ti,ab. (2)

18 architect stat.ti,ab. (94)

19 accutni*.ti,ab. (121)

20 accutnl*.ti,ab. (5)

21 centaur ultra.ti,ab. (6)

22 HISCL.ti,ab. (54)

23 vidas.ti,ab. (1014)

For peer review only

24	lumipulse.ti,ab. (170)
25	serial troponin.ti,ab. (193)
26	or/13-25 (17498)
27	exp Emergency Service, Hospital/ (2120)
28	(emergency adj (room* or department*)).ti,ab. (133487)
29	(ER or ED).ti,ab. (194228)
30	(presenting or presented).ti,ab. (1889770)
31	presentation.ti,ab. (428459)
32	(admission or admitted).ti,ab. (477276)
33	chest pain unit.ti,ab. (349)
34	or/27-33 (2737124)
35	12 and 26 and 34 (3268)
36	limit 35 to yr="2006 -Current" (2862)

For peer review only

Appendix 2 Data extraction form

General information:

Author

Title

Journal (incl. volume and page)

Year of publication

Institution

Country

Language

Translation details (if applicable)

Funding

Ethical approval

Characteristics of the study:

Study design

- Prospective vs. retrospective
- Single-gate vs. two-gate
- Multi-test design vs. randomized design vs. non-comparative design (i.e., single test)
- Multi-centre vs. single centre

Inclusion criteria

Exclusion criteria

Description of clinical setting, referral and selection process

Study years

Number of participants:

Eligible

Enrolled

Completed index test

Completed reference standard

Analysed

Excluded/dropped out (plus reasons)

Participants' characteristics:

Age: mean (SD)/median (IQR), range

For peer review only

Sex (n/% of male patients enrolled)

Time from symptom onset to presentation: mean (SD)/median (IQR), range

Risk score (e.g., TIMI, HEART, NCPR, GRACE, and etc.)

Other relevant information (smoking status, hypertension, diabetes, dyslipidaemia, prior history of MI/AP)

Prior tests (symptoms, vital signs, ECG)

Index test:

Assay

Description of decision rule (if applicable)

Cut-off values for positive disease

Cut-off values for negative disease

Blinding to the reference standard

Blinding to other clinical information

Reference standard:

Definition of MI

Single vs. composite vs. any rule vs. expert consensus

Assessors

Number (kappa if multiple assessors involve)

Experience

Blinding to the index test

Blinding to other clinical information

Test accuracy data

Number of TPs, FPs, FNs, TNs

Reported sensitivity, specificity, PPV, NPV

ROC curve (if reported)

For peer review only

Appendix 3 Criteria for rating the methodological quality of the included studies based on the QUADAS-2 tool

1. Domain: Patient selection
    a. Risk of bias

Question 1: Was a consecutive or random sample of patients enrolled? 'Yes' if consecutive or random sampling explicitly stated and described in sufficient detail; 'No' if non-consecutive or convenience sampling was used, and 'Unclear' if no sufficient information was provided to make a decision.

Question 2: Did the study avoid inappropriate exclusions? 'Yes' if all patients suspected of ACS that would normally undergo cardiac troponin testing were included; 'No' if relevant patient groups (e.g. patients with no history of CAD) were excluded and 'Unclear' if the reported data did not allow to make a judgement.

    b. Concerns regarding applicability

Are there concerns that the included patients and setting do not match the review question? 'No' if unselected patients >18 years of age presenting to the ED with symptoms suggestive of non-ST-segment elevation myocardial infarction included; 'Yes' if the patients or the setting did not match the review question (e.g. patients with ST-segment elevation included); and 'Unclear' if unspecified.

2. Domain: Index test
    a. Risk of bias

Question 1: If a threshold was used, was it pre-specified? 'Yes' if the results for at least one pre-specified threshold were reported; 'No' if ROC-optimisation or other methods were used to define a threshold.

    b. Concerns regarding applicability

Are there concerns that the index test, its conduct, or its interpretation differ from the review question? 'No' if the assay was used according to the manufacturer's recommendations; 'Yes' otherwise.

3. Domain: Reference standard
    a. Risk of bias

Question 1: Is the reference standard likely to classify correctly the target condition? 'Yes' if the diagnosis of myocardial infarction was adjudicated independently by two qualified clinicians according to the current universal definition (Thygesen 2012) and the method included high-sensitivity troponin assay; 'No' if not (including studies using an earlier generation of troponin assay or other biomarker as a reference test for myocardial necrosis) and 'Unclear' if not reported in sufficient detail.

Question 2: Was the reference standard independent from the index test (i.e. the index test did not form part of the reference standard)? 'Yes' if different troponin assay was used; 'No' if the results from the index test were included in the reference standard; and 'Unclear' if not reported in sufficient detail.

Question 3: Were the results from the reference standard interpreted without knowledge of the results from the index test? 'Yes' or 'No' if explicitly stated, 'Unclear' if not reported.

For peer review only

b. Concerns regarding applicability

Are there concerns that the target condition as defined by the reference standard does not match the review question? 'No' if the target condition was Non-ST-segment Elevation Myocardial Infarction (NSTEMI), diagnosed according to the current universal definition; 'Yes' if patients with ST-segment elevation on the initial ECG were included or the target condition deviated in some other way from the above definition.

4. Domain: Flow and timing
   a. Risk of bias

Question 1: Was there an appropriate interval between the index test and reference standard? 'Yes' if the reference test was done within 24 hours of the index test; 'No' if more than 24 hours and 'Unclear' if not reported.

Question 2: Did all patients receive a reference standard? 'Yes' if all patients included in the analysis received a reference standard; 'No' if some patients were included in the analysis without receiving a reference standard; 'Unclear' otherwise.

Question 3: Did all patients receive the same reference standard? 'Yes' if all patients received the same reference standard; 'No' otherwise and 'Unclear' if insufficient information.

Question 4: Were all patients included in the analysis? 'Yes' if all patients enrolled in the study were accounted for and included in the analysis; 'No' if patients who were enrolled in the study were excluded from the analysis, and 'Unclear' if insufficient data were available to decide.

Rules for producing an overall risk of bias rating for each domain:

• If all signalling questions within a domain are answered 'Yes' the overall domain rating is 'Low risk of bias'

• If one or more signalling questions within a domain are answered 'No' the overall domain rating is 'High risk of bias'

• If one or more signalling questions in the domain are answered 'Unclear' while the remaining signalling questions are answered 'Yes' then the overall domain rating is 'Unclear risk of bias'.

For peer review only

Reporting checklist for protocol of a systematic review.

Based on the PRISMA-P guidelines.

Instructions to authors

Complete this checklist by entering the page numbers from your manuscript where readers will find each of the items listed below.

Your article may not currently address all the items on the checklist. Please modify your text to include the missing information. If you are certain that an item does not apply, please write "n/a" and provide a short explanation.

Upload your completed checklist as an extra file when you submit to a journal.

In your methods section, say that you used the PRISMA-P reporting guidelines, and cite them as:

Moher D, Shamseer L, Clarke M, Ghersi D, Liberati A, Petticrew M, Shekelle P, Stewart LA. Preferred Reporting Items for Systematic Review and Meta-Analysis Protocols (PRISMA-P) 2015 statement. Syst Rev. 2015;4(1):1.

		Reporting Item	Page Number
Identification	#1a	Identify the report as a protocol of a systematic review	1
Update	#1b	If the protocol is for an update of a previous systematic review, identify as such	n/a
	#2	If registered, provide the name of the registry (such as PROSPERO) and registration number	4
Contact	#3a	Provide name, institutional affiliation, e-mail address of all protocol authors; provide physical mailing address of corresponding author	1-2
Contribution	#3b	Describe contributions of protocol authors and identify the guarantor of the review	18
	#4	If the protocol represents an amendment of a previously completed or published protocol, identify as such and list changes; otherwise, state plan for documenting important protocol amendments	18

For peer review only

Sources	#5a	Indicate sources of financial or other support for the review	18-19
Sponsor	#5b	Provide name for the review funder and / or sponsor	18-19
Role of sponsor or funder	#5c	Describe roles of funder(s), sponsor(s), and / or institution(s), if any, in developing the protocol	19
Rationale	#6	Describe the rationale for the review in the context of what is already known	9-11
Objectives	#7	Provide an explicit statement of the question(s) the review will address with reference to participants, interventions, comparators, and outcomes (PICO)	11
Eligibility criteria	#8	Specify the study characteristics (such as PICO, study design, setting, time frame) and report characteristics (such as years considered, language, publication status) to be used as criteria for eligibility for the review	11-13
Information sources	#9	Describe all intended information sources (such as electronic databases, contact with study authors, trial registers or other grey literature sources) with planned dates of coverage	14
Search strategy	#10	Present draft of search strategy to be used for at least one electronic database, including planned limits, such that it could be repeated	Supplementary file
Study records - data management	#11a	Describe the mechanism(s) that will be used to manage records and data throughout the review	14
Study records - selection process	#11b	State the process that will be used for selecting studies (such as two independent reviewers) through each phase of the review (that is, screening, eligibility and inclusion in meta-analysis)	14
Study records - data collection process	#11c	Describe planned method of extracting data from reports (such as piloting forms, done independently, in duplicate), any processes for obtaining and confirming data from investigators	14-15
Data items	#12	List and define all variables for which data will be	14-15,

For peer review only

		sought (such as PICO items, funding sources), any pre-planned data assumptions and simplifications	Supplementary file
Outcomes and prioritization	#13	List and define all outcomes for which data will be sought, including prioritization of main and additional outcomes, with rationale	13
Risk of bias in individual studies	#14	Describe anticipated methods for assessing risk of bias of individual studies, including whether this will be done at the outcome or study level, or both; state how this information will be used in data synthesis	15
Data synthesis	#15a	Describe criteria under which study data will be quantitatively synthesised	16
	#15b	If data are appropriate for quantitative synthesis, describe planned summary measures, methods of handling data and methods of combining data from studies, including any planned exploration of consistency (such as I2, Kendall's τ)	16-17
	#15c	Describe any proposed additional analyses (such as sensitivity or subgroup analyses, meta-regression)	17
	#15d	If quantitative synthesis is not appropriate, describe the type of summary planned	17
Meta-bias(es)	#16	Specify any planned assessment of meta-bias(es) (such as publication bias across studies, selective reporting within studies)	15
Confidence in cumulative evidence	#17	Describe how the strength of the body of evidence will be assessed (such as GRADE)	n/a

The PRISMA-P checklist is distributed under the terms of the Creative Commons Attribution License CC-BY 4.0. This checklist can be completed online using https://www.goodreports.org/, a tool made by the EQUATOR Network in collaboration with Penelope.ai

For peer review only

BMJ Open

Diagnostic accuracy of contemporary and high-sensitivity cardiac troponin assays used in serial testing, versus single-sample testing as a comparator, to triage patients suspected of acute non-ST-segment elevation myocardial infarction (NSTEMI): A systematic review protocol

Journal:	*BMJ Open*
Manuscript ID	bmjopen-2018-026012.R1
Article Type:	Protocol
Date Submitted by the Author:	26-Nov-2018
Complete List of Authors:	Zhelev, Zhivko; University of Exeter, Exeter Test Group, University of Exeter Medical School<br>Ohtake, Hirotaka; Fujita Health University School of Medicine, Department of Emergency and General Internal Medicine<br>Iwata, Mitsunaga; Fujita Health University School of Medicine, Department of Emergency and General Internal Medicine<br>Terasawa, Teruhiko ; 1Fujita Health University School of Medicine, Department of Emergency and General Internal Medicine<br>Rogers, Morwenna; University of Exeter Medical School, NIHR CLAHRC South West Peninsula<br>Peters, Jaime; University of Exeter, Exeter Test Group, University of Exeter Medical School<br>Hyde, Chris; University of Exeter, Exeter Test Group, University of Exeter Medical School
**Primary Subject Heading**:	Cardiovascular medicine
Secondary Subject Heading:	Diagnostics, Emergency medicine, Evidence based practice
Keywords:	acute coronary syndrome, Myocardial infarction < CARDIOLOGY, troponin, emergency department, meta-analysis, sesnsitivity and specificity

SCHOLARONE™
Manuscripts

Title: Diagnostic accuracy of contemporary and high-sensitivity cardiac troponin assays used in serial testing, versus single-sample testing as a comparator, to triage patients suspected of acute non-ST-segment elevation myocardial infarction (NSTEMI): A systematic review protocol

Corresponding author:

Full name: Zhivko Zhelev

Postal address: Exeter Test Group, PenCLAHRC
College of Medicine and Health
University of Exeter
South Cloisters (Room 3.09)
St Luke's Campus
EXETER EX1 2LU

E-mail: Z.Zhelev@exeter.ac.uk

Tel: 0044 (0)1392 726093

Co-authors:

Hirotaka Ohtake, MD; Department of Emergency and General Internal Medicine, Fujita Health University School of Medicine, Toyoake, Japan

Mitsunaga Iwata, MD, PhD; Department of Emergency and General Internal Medicine, Fujita Health University School of Medicine, Toyoake, Japan

Teruhiko Terasawa MD PhD, Department of Emergency and General Internal Medicine, Fujita Health University School of Medicine, Toyoake, Japan

For peer review only

Morwenna Rogers, PenCLAHRC, University of Exeter Medical School, University of Exeter, Exeter, UK

Jaime Peters PhD, Exeter Test Group, University of Exeter Medical School, University of Exeter, Exeter, UK

Prof Christopher Hyde, MD PhD, Exeter Test Group, University of Exeter Medical School, University of Exeter, Exeter, UK

For peer review only

Word count: 3875

For peer review only - http://bmjopen.bmj.com/site/about/guidelines.xhtml

ABSTRACT

Introduction

Although the new generation of cardiac troponin assays have revolutionised diagnosis of myocardial infarction (MI), their application in triaging patients with suspected acute coronary syndrome requires further investigation. The objectives of the current systematic review are to evaluate the diagnostic accuracy of contemporary and high-sensitivity cardiac troponin assays used in serial testing, versus single-sample testing as a comparator, to identify patients with non-ST-segment-elevation MI in the emergency department.

Methods and analysis

We will conduct systematic searches of MEDLINE, EMBASE, Science Citation Index, the Cochrane Database of Systematic Reviews and the CENTRAL database covering the period from 1st January 2006 to present, with no restrictions on language or publication status. Two review authors will independently screen studies for inclusion, extract data from eligible studies and assess their methodological quality using QUADAS-2. Studies will be included if they evaluate contemporary or high-sensitivity cardiac troponin assays used in serial testing, in patients presenting to the ED with suspicion of MI. Estimates of sensitivity and specificity from each study will be presented in forest plots and in the receiver operating characteristics space. If appropriate, we will pool the results using Bayesian hierarchical models that allow correction for imperfect reference standard. We will obtain summary estimates of sensitivity and specificity of alternative testing protocols and compare their accuracy. We will also investigate the impact of pre-specified sources of heterogeneity and methodological quality items. If pooling of results is considered inappropriate, we will present our findings in tables and diagrams and will describe them narratively.

For peer review only - http://bmjopen.bmj.com/site/about/guidelines.xhtml

Ethics and dissemination

No formal ethical approval will be sought, but we will report on the ethical approval of the included studies. Dissemination of findings will be through publications in peer-reviewed journals, presentations at conferences and the web-sites of the universities.

PROSPERO registration number: CRD42018106379

Keywords: acute coronary syndrome, myocardial infarction, troponin, emergency department, systematic review, meta-analysis, sensitivity and specificity

ARTICLE SUMMARY

Strengths and limitations of this study

- Systematic and comprehensive searches to identify all published and unpublished studies
- Robust procedures for screening, data extraction and methodological quality assessment, based on pre-specified criteria
- Statistical methods for pooling results that allow correction for an imperfect reference standard
- If the number of studies is too small and heterogeneity in study-level estimates is considered too great, we may not be able to obtain summary estimates of sensitivity and specificity.

For peer review only

INTRODUCTION

Coronary heart disease is one of the most common chronic conditions causing more than 7 million deaths worldwide each year [1]. The underlying pathogenesis is progressive accumulation of atheromatous plaque on the walls of the coronary arteries which, in its advanced stages, obstructs the normal blood flow and causes myocardial ischemia. If the plaque gets ruptured, clots may form and limit, or completely cut off, the blood supply to part of the heart muscle. This life-threatening condition is known as 'acute coronary syndrome' and presents with chest pain, dyspnoea, nausea and other unspecific symptoms. It includes type 1 myocardial infarction (MI), if there is evidence of permanent damage to the heart muscle, or unstable angina, if no evidence of myocardial necrosis is detected [2]. Myocardial infarction could also happen in the absence of acute coronary syndrome, as a result of other processes, including mismatch between myocardial oxygen supply and demand (type 2 MI) [3].

Patients with MI require urgent specialist treatment and their outcomes depend on a timely and accurate diagnosis. However, diagnosis in an emergency setting could be challenging as clinical symptoms lack sufficient sensitivity and specificity [4] and a normal electrocardiogram (ECG) does not rule out the condition. Therefore, in patients suspected of MI but with non-diagnostic ECG, the current guidelines recommend determination of troponin T or I, highly specific biomarkers of cardiac necrosis, both as a triage tool and in establishing the final diagnosis [5 ,6].

Although cardiac troponins are specific to myocardial necrosis, they are not specific to MI and elevated concentration could be found in a number of other acute and chronic conditions that cause myocardial injury. The main difference between acute MI and most of

For peer review only

the other conditions is the pattern of dynamic changes. Acute MI is characterised by a sharp increase in the bloodstream concentration of troponin in the first 24 hours of symptom onset, reflecting the progressive nature of the condition. Consequently, a significant difference in troponin concentrations from two consecutive samples (usually referred to as 'delta troponin') is interpreted as an indication that the most likely underlying condition is acute MI. Another consideration is the fact that in the first two hours of symptom onset the level of cardiac troponin in the bloodstream may not exceed the 99th centile, which is an arbitrary cutoff used to dichotomise patients as having or not having myocardial injury. Therefore, a single determination early in the diagnostic process might miss patients with an evolving MI.

Contemporary and high-sensitivity cardiac troponin assays

Older generations of cardiac troponin assays lacked the precision and sensitivity to identify a small increase in the bloodstream level of the biomarker soon after onset of symptoms. Therefore, testing was done 6 – 12 hours after presentation to the ED, causing considerable delay in diagnosis and treatment, and unnecessary hospital admissions of patients without MI [7]. In the past ten years contemporary and high-sensitivity cardiac troponin assays have been developed and adopted in clinical practice. They can measure small concentrations of troponin with high levels of precision, which means that both patients at high and low risk of having an MI could be identified much earlier in the triage.

Given the large number of cardiac troponin assays marketed as 'high-sensitivity', recent efforts have been made to define criteria that could inform policy and clinical decisions. In the development of their guidance on early rule out of acute MI, [5] the UK's National Institute for Health and Care Excellence (NICE) defined a high-sensitivity cardiac troponin

For peer review only - http://bmjopen.bmj.com/site/about/guidelines.xhtml

assay as one with coefficient of variation (CV) ≤10% at the 99th centile and ability to measure concentrations in at least 50% of the reference population. The systematic review that informed the guidance identified only three commercially available assays that met the above criteria: the Elecsys Troponin T high-sensitive assay (Roche Diagnostics), the ARCHITECTSTAT high sensitive troponin I (Abbott Laboratories) and the AccuTnI+3 (Beckman Coulter) [7]. More recently, the Academy of the American Association for Clinical Chemistry and the Task Force on Clinical Applications of Cardiac Bio-Markers of the International Federation of Clinical Chemistry and Laboratory Medicine have endorsed the above definition of high-sensitivity cardiac troponin assay and have recommended "that assays unable to detect cTn [cardiac troponin] at concentrations at or above the LoD [limit of detection] in at least 50% of healthy men and women be labeled as contemporary cTn [cardiac troponin] assays."(p. 6)[8].

Serial testing

Serial measurement of cardiac troponin is employed to increase both sensitivity and specificity of the diagnostic strategy. This usually involves one sample taken at presentation to the ED and another sample taken one or more hours later. The results from these two determinations and their difference ('delta troponin') are combined in a diagnostic rule to determine the likelihood of the symptoms being caused by an acute MI. The strategy takes advantage of the fact that in the first 24 hours of the onset of symptoms the concentration of cardiac troponin in the bloodstream is progressively rising. According to the current universal definition, diagnosis of MI requires that the patient's presentation is consistent with cardiac ischaemia; cardiac troponin concentration is above the 99th centile of the

For peer review only - http://bmjopen.bmj.com/site/about/guidelines.xhtml

reference population and two consecutive determinations a few hours apart show significant change (e.g. >20%) in cardiac troponin level [9]. Such patients require urgent reperfusion to restore blood flow to the affected part of the heart and should immediately be admitted to hospital.

Clinical pathway

Figure 1 shows the clinical pathway for triaging patients suspected of MI, including single-sample and two-sample cardiac troponin algorithms we identified in our scoping searches. Most patients with symptoms suggestive of acute coronary syndrome are transported by ambulance or present directly to the ED [10]. Their initial triage includes clinical assessment, medical history and a 12-lead ECG. Patients with ST-segment elevation MI (STEMI) or other intermediate- or high-risk features are directly admitted to hospital. Low risk patients undergo troponin testing, with the first sample taken at presentation and the second sample one or more hours later. If both determinations are below a pre-specified clinical cutoff, e.g. the 99th centile, and there is no evidence of evolving MI (e.g. rising troponin, new angina or other symptoms), the patient is diagnosed with unstable angina or alternative explanation of the symptoms is sought.

Diagnostic strategies used in the triage of patients suspected of non-ST-segment elevation MI (NSTEMI)

Taking advantage of the high sensitivity and precision of the new generation cardiac troponin assays, a range of diagnostic protocols addressing specific clinical scenarios have been devised. They could be summarised as follows:

For peer review only - http://bmjopen.bmj.com/site/about/guidelines.xhtml

- Single-sample protocols to identify patients at low risk of MI: If the concentration of troponin in the first sample, taken two or more hours after the onset of symptoms, is well below the 99th centile (e.g. below the limit of detection or other pre-specified cutoff), the probability of MI is also very low and the patient could be discharged, if appropriate, or alternative explanation of the symptoms could be sought. For instance, a meta-analysis conducted by Chapman and colleagues [11] showed that the negative predictive value of Abbott ARCHITECT$_{STAT}$ high-sensitive cardiac troponin I assay (Abbott Laboratories) is 99.5%, when the cutoff is "< 5 ng/L"; the 99th centile of this assay is much higher: 34 ng/L for men and 16 ng/L for women [12]. Alternative early low-risk strategies are also been proposed, e.g. combining a single sample high-sensitivity troponin with copeptin [13 ,14] or information from the ECG and risk assessment tools, such as the Thrombolysis in Myocardial Infarction (TIMI) risk score [15 ,16]. This increases the negative predictive value of the protocol and the proportion of patients assigned to the low-risk group.
- Two-sample protocols to identify patients at low risk of MI:
    - If the results from two samples taken one or more hours apart are below a pre-specified threshold, such as the 99th centile or lower, and there is no significant change, the probability of MI is low.
    - If both values are slightly above the 99th centile, but there is no change, the underlying cause might be a chronic condition, such as heart failure or myocarditis.
- Single-sample protocols to identify patients at high risk of MI: In the context of myocardial ischemia, if the concentration of troponin in the first sample is well

For peer review only - http://bmjopen.bmj.com/site/about/guidelines.xhtml

above the 99th centile, the probability of MI is high and the patient will most likely be admitted to hospital for further testing and treatment.

- Two-sample rule-in: The universal diagnosis of MI requires that at least one of two consecutive samples taken three or more hours apart produces a value above the 99th centile and there is a significant change in the concentration of cardiac troponin between the first and the second sample [9].

The above protocols could be combined in complex diagnostic strategies that use different cutoffs to identify patients at low, intermediate or high risk of MI. Such strategies enable clinicians to stratify patients early in the triage, so that appropriate referral and treatment decisions could be made without delay.

Rationale for conducting the review

Recent meta-analyses have focused on single-sample protocols designed to identify patients at low risk of MI [11 ,17 ,18]. Such protocols have important implications for clinical practice, especially in terms of cost-effectiveness and quality of patient care: they could free up capacity required for patients who need emergency treatment; reduce the number of unnecessary tests and the associated risks; the focus of clinical investigation could shift earlier to an alternative explanation of the patient's symptoms and, if appropriate, the patient could be discharged directly from the ED [10].

However, single-sample protocols are limited in their application, as many patients without MI will present with troponin concentrations above the pre-specified cutoff. Such patients will undergo a second troponin determination which, if the inter-sample time is three or more hours, as recommended in the current NICE guidance,[5] will result in the patients

For peer review only - http://bmjopen.bmj.com/site/about/guidelines.xhtml

spending many hours in hospital. In some cases they will have to stay overnight, until a qualified clinician is available to review results and make a decision for transfer or discharge.

Various serial testing protocols have been devised and validated, including protocols using different cutoffs to identify patients at low and high risk of MI and shorter (one or two hours) inter-sample time. Such protocols could enable discharge or referral decisions to be made for a larger proportion of patients while still in the ED. A brief scoping of the literature showed considerable variation in these protocols and their accuracy, and we can assume that the variation in clinical practice is even greater. The differences concern:

- The analytical and diagnostic performance of different assays
- The cutoffs used to identify patients at low and high risk of MI
- The method of calculating delta troponin: absolute versus relative change (e.g. >4.5 ng/L vs. >20%), and
- The inter-sample time (1, 2 or more hours).

Other factors, such as the time from onset of symptoms, patient characteristics (age, sex, risk factors) and prior tests (e.g. ECG findings) are also likely to affect the performance of the assays and contribute to the observed heterogeneity in study-level estimates [11]. This could be confusing when making decisions about which protocol to implement in practice, in order to achieve the best balance between accuracy and efficiency (the proportion of patients for whom a decision for discharge or admission to a specialist unit could be made early in the triage).

Therefore, we are proposing to systematically identify, review and summarise research evidence pertaining to the diagnostic accuracy of contemporary and high-sensitivity cardiac troponin assays, when used in serial testing to identify MI in symptomatic patients

For peer review only - http://bmjopen.bmj.com/site/about/guidelines.xhtml

presenting to the ED. Our primary objective will be to obtain summary estimates of the accuracy of specific protocols and the proportion of patients assigned to each risk category. Our secondary objectives will be to determine the relative accuracy of alternative protocols, including a direct comparison of single-sample and two-sample protocols; and to investigate the impact of protocol- and study-level characteristics on test accuracy estimates.

METHODS AND ANALYSIS

In preparing the protocol, we used the PRISMA-P reporting guidelines [19].

Eligibility criteria

Study design

We will include all primary diagnostic accuracy studies with single-gate design (also known as diagnostic cohort studies). Studies with two-gate design will be excluded due to inherent bias in the reported diagnostic accuracy estimates [20].

Participants

Only studies in patients 18 years of age or older, who present to the ED with suspicion of acute coronary syndrome, will be considered for inclusion. Studies in patients with STEMI will be excluded, but we will include studies analysing jointly STEMI and non-STEMI patients. We will include only data for non-STEMI, if such data could be obtained; otherwise, we will deal with this in the methodological quality assessment and the sensitivity analysis.

Index tests

The index tests are contemporary or high-sensitivity cardiac troponin assays used in serial measurement (two or more determinations) to identify MI. To qualify as 'contemporary' or

For peer review only

For peer review only - http://bmjopen.bmj.com/site/about/guidelines.xhtml

'high-sensitivity' assays need to have CV ≤10% at the 99th percentile of normal; 'contemporary' assays should be able to detect cardiac troponin in 20% to <50% of healthy individuals and 'high-sensitivity' assays in at least 50%. We will include all cardiac troponin assays that meet the above criteria. We will ascertain eligibility by checking package inserts of the assays, contacting manufacturers and identifying published clinical evaluations.

Target conditions and reference standard

Our primary target condition will be an index diagnosis of MI. Our secondary target conditions will be:

- A composite outcome of index MI and cardiac death within 30 days of the index presentation.
- A composite outcome of index MI, death and major adverse cardiac events (MACEs) including cardiac arrest, emergency revascularization procedure, cardiogenic shock, ventricular arrhythmia or high-degree atrioventricular block requiring intervention, if they occur within 30 days of the index presentation.

The reference standard is a final diagnosis of MI adjudicated by qualified clinicians according to the universal definition of MI [9]. The quality of the reference standard, such as independence from the index test, adjudication and blinding to the index test results, will be considered in the methodological quality assessment.

Outcomes

The main outcomes are test accuracy data reported as true positives, false positives, false negatives and true negatives for protocols with dichotomous outcomes, or three-by-two tables for protocols that assign patients to low, intermediate and high risk categories. If the

For peer review only - http://bmjopen.bmj.com/site/about/guidelines.xhtml

contingency table is not reported in the paper, we will derive data from the sensitivity and specificity estimates, the total number of patients and the proportion of those with the target condition. Studies that report only test accuracy estimates but insufficient detail to reconstruct the original contingency table will be included in the review, but will be excluded from the quantitative analysis. Studies that meet the rest of the criteria, but do not report test accuracy data, will be excluded only after we have tried but failed to obtain the missing data.

Publication status and language

We will include conference abstracts and unpublished studies if they meet our eligibility criteria and we are able to obtain the necessary data. We will include all relevant papers regardless of the language in which they are published. If necessary, we will arrange translation or data extraction by a person fluent in the respective language.

Searches

We plan to perform systematic searches of the following electronic databases: MEDLINE (via OvidSp), EMBASE (via OvidSP), Science Citation Index (via Web of Science), Cochrane Database of Systematic Reviews and CENTRAL database (via the Cochrane Library). We will search all databases from January 1st 2006 to present, with no restrictions on language or publication status. As far as we are aware, no studies evaluating the diagnostic accuracy of high-sensitivity cardiac troponin assays have been published prior to 2010 (Zhelev 2015, Westwood 2015, Chapman 2017, Pickering 2017). Hence, the choice of the above publication date as a cutoff for our searches, allowing for some uncertainty in the exact date.

For peer review only - http://bmjopen.bmj.com/site/about/guidelines.xhtml

Related systematic reviews will be examined for additional studies for inclusion. Our search strategy complies with the Peer Review of Electronic Search Strategies (PRESS)-2015 Guideline for developing electronic searches. It was developed by an information specialist (MR) with many years of experience of using databases for systematic reviews and combines terms for the target condition, index tests and setting. The search strategy for Embase is provided as Appendix 1 in the Supplemental file, and the search strategies for the other databases could be obtained from the authors upon request.

In addition, we will conduct forward and backward searches of all included papers and other relevant publications (e.g. previous systematic reviews), and will contact experts in the field and study authors to check for missed titles or unpublished data.

Selection of studies for inclusion

The results from the electronic searches will be imported into a reference management software and de-duplicated. Two review authors will conduct all screening independently with disagreements resolved through discussion or arbitration. First, we will screen all titles and abstracts; the full text of any potentially relevant publications will be retrieved and assessed for eligibility against our inclusion criteria. The same procedure will be repeated for titles identified in the additional searches.

Data extraction

We have developed and piloted a data extraction form available in the online supplement (Appendix 2 in the Supplementary file). Data will be extracted for the following topics: publication details, study characteristics, patients, index tests, reference standard, outcomes and methodological quality items. Two review authors will extract data

For peer review only - http://bmjopen.bmj.com/site/about/guidelines.xhtml

independently and all disagreements will be resolved through discussion or arbitration. Study authors will be contacted for missing data and to ascertain the independence of study cohorts, with reminders sent two weeks after the initial request.

Methodological quality assessment

We will assess the methodological quality of the included studies using a tailored version of the Quality Assessment of Diagnostic Accuracy Studies version 2 (QUADAS-2) tool. The tool consists of four domains: Patient selection, Index test, Reference standard and Flow and timing. Each domain is assessed for risk of bias and rated as “High risk”, “Low risk” or “Unclear risk”. In addition, the first three domains are assessed for applicability concerns and rated using the same categories. The authors of the tool recommend that it is tailored for each systematic review by adding or removing signalling questions [21]. A tailored version of the tool with details of the signalling questions, operational definitions and the rules for combining the answers to produce a domain-level rating is included in the Supplementary file (Appendix 3).

Assessment of publication bias

Given the limited knowledge of publication bias in diagnostic accuracy studies and the controversies around its assessment, [22 ,23] we will not use statistical methods to detect the presence of publication bias. We acknowledge, however, that publication bias might be present and have bearing on our findings. We will try to mitigate this by conducting comprehensive searches of the published research, actively seek to identify unpublished data and note the possibility of publication bias when reporting our findings.

Statistical analysis and data synthesis

For peer review only - http://bmjopen.bmj.com/site/about/guidelines.xhtml

Contingency tables will be reconstructed from the data reported in the papers or by entering sensitivity, specificity, total number of patients and the proportion of those with the target condition in the test accuracy calculator of the Cochrane Collaboration's Review Manager 5.3 (The Nordic Cochrane Centre, The Cochrane Collaboration, 2014). Sensitivity and specificity estimates with 95% confidence intervals will be calculated and presented in forest plots and in the receiver operating characteristics (ROC) space. Data will be grouped by individual assays and protocols. We will conduct visual inspection of each plot, to determine the level and potential sources of heterogeneity, and to decide whether meta-analysis is appropriate.

For each assay, if a sufficient number of studies per protocol are available and the level of clinical and statistical between-study heterogeneity is low, we will conduct separate meta-analyses by dichotomising the results from studies reporting more than two outcomes (e.g. low, intermediate and high-risk categories). First, we will combine the intermediate and high-risk categories, to obtain a two-by-two table for the low-risk rule of the protocol. Then, we will combine the low and intermediate-risk categories, to obtain a two-by-two table for the high-risk rule of the protocol. These will be included in separate meta-analyses, to obtain summary sensitivity and specificity for the low and high-risk part of the protocol.

Since both the index tests and the reference standard are cardiac troponin assays for the detection of myocardial necrosis, we assume the presence of incorporation bias and will pool the results using the model developed by Dendukuri and colleagues [24]. The model, an extension of the hierarchical summary receiver operating characteristic (HSROC) model, allows correction for imperfect reference standard assuming an underlying continuous latent variable. The same methods will also be used to investigate the comparative accuracy

For peer review only - http://bmjopen.bmj.com/site/about/guidelines.xhtml

of alternative assays and protocols, and the impact of protocol- and study-level characteristics. As a sensitivity analysis, we will also analyse the data using the HSROC model where incorporation bias is not accounted for.

If we decide that pooling of results would be inappropriate (for instance, because the number of studies is too small and heterogeneity too great), we will summarise the results in tables and graphs, and will provide a narrative summary of the observed differences.

Investigation of heterogeneity:

The protocol- and study-level-characteristics explored will include the following potential sources of heterogeneity:

- Definition of the target condition: type 1 MI vs. type 1 and type 2 MI
- Different inter-sample time
- Age (≤65 or >65 years) and sex
- Time since symptom onset (≤2 or >2 hours)
- Presence of myocardial ischemia on electrocardiogram, e.g. ST-segment changes or T-wave inversion
- History of ischemic heart disease.

Sensitivity analysis

If appropriate, we will conduct sensitivity analysis by excluding studies of poor methodological quality and the most influential studies, to assess the robustness of the summary estimates.

ETHICS AND DISSEMINATION

For peer review only - http://bmjopen.bmj.com/site/about/guidelines.xhtml

As this is a systematic review, we are not planning to obtain a formal ethical approval. However, we will extract and report data on the ethical approval of the included studies. The findings from the review will be disseminated through publications in peer-reviewed journals, presentations at conferences and using the web sites and networks of the two universities. Changes and amendments to the protocol will be acknowledged in all presentations and publications.

AUTHOR CONTRIBUTIONS

TT originated the idea; TT and ZZ drafted the initial version of the protocol; MR developed the search strategy and wrote the section on search methods; CH, JP, HO and MI reviewed the protocol and suggested amendments. All authors read and approved the final version of the protocol. TT and ZZ are guarantors of the review.

COMPETING INTERESTS STATEMENT

None declared.

PATIENT AND PUBLIC INVOLVEMENT

No patients have been involved in the development of the protocol.

ACKNOWLEDGEMENTS

None.

FUNDING

This research was funded in part by Fujita Health University, Japan, the Ministry of Education, Culture, Sports, Science and Technology (MEXT), Japan (grant numbers: 18K08902 and 26460755), and the National Institute for Health Research (NIHR)

For peer review only - http://bmjopen.bmj.com/site/about/guidelines.xhtml

Collaboration for Leadership in Applied Health Research and Care South West Peninsula (NIHR CLAHRC South West Peninsula). The views expressed are those of the author(s) and not necessarily those of the Fujita Health University, the MEXT, the NHS, the NIHR or the Department of Health and Social Care. The funders have no role in the development of the protocol.

DATA SHARING

Dataset will be published as a supplementary file.

REFERENCES

1. World Health Organization. Cardiovascular diseases (CVDs): Key facts. 17 May 2017. http://www.who.int/news-room/fact-sheets/detail/cardiovascular-diseases-(cvds) (date accessed 6 Aug 2018).
2. Ibanez B, James S, Agewall S, et al. 2017 ESC Guidelines for the management of acute myocardial infarction in patients presenting with ST-segment elevation. The Task Force for the management of acute myocardial infarction in patients presenting with ST-segment elevation of the European Society of Cardiology (ESC). *European Heart Journal* 2018;**39**(2):119-77.
3. McCarthy CP, Vaduganathan M, Januzzi JL, et al. Type 2 myocardial infarction—diagnosis, prognosis, and treatment. *JAMA* 2018;**320**(5):433-34.
4. Bruyninckx R, Aertgeerts B, Bruyninckx P, et al. Signs and symptoms in diagnosing acute myocardial infarction and acute coronary syndrome: a diagnostic meta-analysis. *The British journal of general practice : the journal of the Royal College of General Practitioners* 2008;**58**(547):105-11.
5. The National Institute for Health and Care Excellence. Myocardial infarction (acute): Early rule out using high-sensitivity troponin tests (Elecsys Troponin T high-sensitive, ARCHITECT STAT High Sensitive Troponin-I and AccuTnI+3 assays): Diagnostics guidance [DG15]. 2014. https://www.nice.org.uk/guidance/dg15 (date accessed 6 Aug 2018).
6. Roffi M, Patrono C, Collet JP, et al. 2015 ESC Guidelines for the management of acute coronary syndromes in patients presenting without persistent ST-segment elevation: Task Force for the Management of Acute Coronary Syndromes in Patients Presenting without Persistent ST-Segment Elevation of the European Society of Cardiology (ESC). *Eur Heart J* 2016;**37**(3):267-315.
7. Westwood M, van Asselt T, Ramaekers B, et al. High-sensitivity troponin assays for the early rule-out or diagnosis of acute myocardial infarction in people with acute chest pain: a systematic review and cost-effectiveness analysis. *Health technology assessment (Winchester, England)* 2015;**19**(44):1-234.
8. Wu AHB, Christenson RH, Greene DN, et al. Clinical Laboratory Practice Recommendations for the Use of Cardiac Troponin in Acute Coronary Syndrome: Expert Opinion from the Academy of the American Association for Clinical Chemistry and the Task Force on Clinical Applications of Cardiac Bio-Markers of the International Federation of Clinical Chemistry and Laboratory Medicine. *Clinical chemistry* 2018;**64**(4):645-55.

For peer review only - http://bmjopen.bmj.com/site/about/guidelines.xhtml

9. Thygesen K, Alpert JS, Jaffe AS, et al. Third universal definition of myocardial infarction. *Circulation* 2012;**126**(16):2020-35.
10. Rachuba S, Salmon A, Zhelev Z, et al. Redesigning the diagnostic pathway for chest pain patients in emergency departments. *Health care management science* 2018;**21**(2):177-91.
11. Chapman AR, Lee KK, McAllister DA, et al. Association of High-Sensitivity Cardiac Troponin I Concentration With Cardiac Outcomes in Patients With Suspected Acute Coronary Syndrome. *Jama* 2017;**318**(19):1913-24.
12. Lee GR, Browne TCA, Guest B, et al. Transitioning high sensitivity cardiac troponin I (hs-cTnI) into routine diagnostic use: More than just a sensitivity issue. *Practical Laboratory Medicine* 2016;**4**:62-75.
13. Balmelli C, Meune C, Twerenbold R, et al. Comparison of the performances of cardiac troponins, including sensitive assays, and copeptin in the diagnostic of acute myocardial infarction and long-term prognosis between women and men. *American heart journal* 2013;**166**(1):30-7.
14. Raskovalova T, Twerenbold R, Collinson PO, et al. Diagnostic accuracy of combined cardiac troponin and copeptin assessment for early rule-out of myocardial infarction: a systematic review and meta-analysis. *Eur Heart J Acute Cardiovasc Care* 2014;**3**(1):18-27.
15. Cullen L, Mueller C, Parsonage WA, et al. Validation of high-sensitivity troponin I in a 2-hour diagnostic strategy to assess 30-day outcomes in emergency department patients with possible acute coronary syndrome. *Journal of the American College of Cardiology* 2013;**62**(14):1242-49.
16. Than M, Cullen L, Aldous S, et al. 2-Hour accelerated diagnostic protocol to assess patients with chest pain symptoms using contemporary troponins as the only biomarker: the ADAPT trial. *Journal of the American College of Cardiology* 2012;**59**(23):2091-8.
17. Pickering JW, Than MP, Cullen L, et al. Rapid rule-out of acute myocardial infarction with a single high-sensitivity cardiac troponin t measurement below the limit of detection: A collaborative meta-analysis. *Annals of internal medicine* 2017;**166**(10):715-24.
18. Zhelev Z, Hyde C, Youngman E, et al. Diagnostic accuracy of single baseline measurement of Elecsys Troponin T high-sensitive assay for diagnosis of acute myocardial infarction in emergency department: systematic review and meta-analysis. *BMJ : British Medical Journal* 2015;**350:h15**.
19. Moher D, Shamseer L, Clarke M, et al. Preferred reporting items for systematic review and meta-analysis protocols (PRISMA-P) 2015 statement. *Systematic reviews* 2015;**4**:1.
20. Rutjes AW, Reitsma JB, Vandenbroucke JP, et al. Case-control and two-gate designs in diagnostic accuracy studies. *Clinical chemistry* 2005;**51**(8):1335-41.
21. Whiting PF, Rutjes AW, Westwood ME, et al. QUADAS-2: a revised tool for the quality assessment of diagnostic accuracy studies. *Annals of internal medicine* 2011;**155**(8):529-36.
22. Deeks JJ, Macaskill P, Irwig L. The performance of tests of publication bias and other sample size effects in systematic reviews of diagnostic test accuracy was assessed. *Journal of clinical epidemiology* 2005;**58**(9):882-93.
23. Macaskill P GC, Deeks JJ, Harbord RM, Takwoingi Y. Chapter 10: Analysing and Presenting Results. In: Deeks JJ BP, Gatsonis C, ed. Cochrane Handbook for Systematic Reviews of Diagnostic Test Accuracy Version 10: The Cochrane Collaboration, 2010.
24. Dendukuri N, Schiller I, Joseph L, et al. Bayesian meta-analysis of the accuracy of a test for tuberculous pleuritis in the absence of a gold standard reference. *Biometrics* 2012;**68**(4):1285-93.

FIGURE LEGENDS

For peer review only - http://bmjopen.bmj.com/site/about/guidelines.xhtml

Figure 1 Clinical pathway for triaging patients suspected of MI, including single-sample and two-sample cardiac troponin algorithms identified in scoping searches

For peer review only

For peer review only - http://bmjopen.bmj.com/site/about/guidelines.xhtml

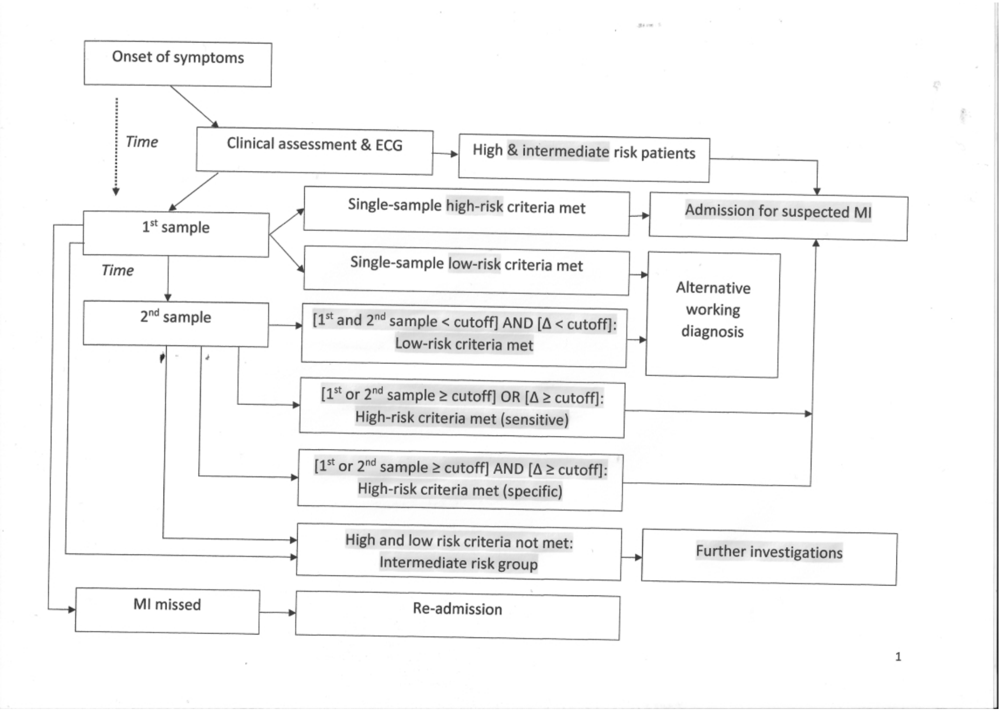

Clinical pathway for triaging patients suspected of MI, including single-sample and two-sample cardiac troponin algorithms identified in scoping searches

296x210mm (300 x 300 DPI)

SUPPLEMENTARY FILE

Appendix 1 Search strategy for Embase

----

1 exp Chest Pain/

2 (chest adj2 pain*).ti,ab.

3 chest discomfort.ti,ab.

4 exp Acute Coronary Syndrome/

5 acute coronary syndrome*.ti,ab.

6 ACS.ti,ab.

7 exp Angina, Unstable/

8 unstable angina.ti,ab.

9 Myocardial Infarction/di

10 myocardial infarction.ti,ab.

11 heart attack.ti,ab.

12 or/1-11

13 cardiac troponin*.ti,ab.

14 high* sensitiv* troponin*.ti,ab.

15 hs-ctn*.ti,ab.

16 elecsys.ti,ab.

17 architectstat.ti,ab.

18 architect stat.ti,ab.

19 accutni*.ti,ab.

20 accutnl*.ti,ab.

21 centaur ultra.ti,ab.

22 HISCL.ti,ab.

23 vidas.ti,ab.

24 lumipulse.ti,ab.

25 serial troponin.ti,ab.

26 contemporary troponin.ti,ab.

27 heart adp.ti,ab.

28 (EDACS and troponin).ti,ab.

29 (Adapt adp and troponin).ti,ab.

30 (GRACE and troponin).ti,ab.

For peer review only

31 (TIMI and troponin).ti,ab.

32 or/13-31

33 exp Emergency Service, Hospital/

34 (emergency adj (room* or department*)).ti,ab.

35 (ER or ED).ti,ab.

36 (presenting or presented).ti,ab.

37 presentation.ti,ab.

38 (admission or admitted).ti,ab.

39 chest pain unit.ti,ab.

40 or/33-39

41 12 and 32 and 40

42 limit 41 to yr="2006 -Current"

For peer review only

Appendix 2 Data extraction form

General information:

Author

Title

Journal (incl. volume and page)

Year of publication

Institution

Country

Language

Translation details (if applicable)

Funding

Ethical approval

Characteristics of the study:

Study design

- Prospective vs. retrospective
- Single-gate vs. two-gate
- Multi-test design vs. randomized design vs. non-comparative design (i.e., single test)
- Multi-centre vs. single centre

Inclusion criteria

Exclusion criteria

Description of clinical setting, referral and selection process

Study years

Number of participants:

Eligible

Enrolled

Completed index test

Completed reference standard

Analysed

Excluded/dropped out (plus reasons)

Participants' characteristics:

Age: mean (SD)/median (IQR), range

For peer review only

Sex (n/% of male patients enrolled)

Time from symptom onset to presentation: mean (SD)/median (IQR), range

Risk score (e.g., TIMI, HEART, NCPR, GRACE, and etc.)

Other relevant information (smoking status, hypertension, diabetes, dyslipidaemia, prior history of MI/AP)

Prior tests (symptoms, vital signs, ECG)

Index test:

Assay

Description of decision rule (if applicable)

Cut-off values for positive disease

Cut-off values for negative disease

Blinding to the reference standard

Blinding to other clinical information

Reference standard:

Assay used to make a diagnosis

Definition of MI

Single vs. composite vs. any rule vs. expert consensus

Assessors

Number (kappa if multiple assessors involve)

Experience

Blinding to the index test

Blinding to other clinical information

Test accuracy data

Number of TPs, FPs, FNs, TNs

Reported sensitivity, specificity, PPV, NPV

ROC curve (if reported)

For peer review only

Appendix 3 Criteria for rating the methodological quality of the included studies based on the QUADAS-2 tool

1. Domain: Patient selection
    a. Risk of bias

Question 1: Was a consecutive or random sample of patients enrolled? 'Yes' if consecutive or random sampling explicitly stated and described in sufficient detail; 'No' if non-consecutive or convenience sampling was used, and 'Unclear' if no sufficient information was provided to make a decision.

Question 2: Did the study avoid inappropriate exclusions? 'Yes' if all patients suspected of ACS that would normally undergo cardiac troponin testing were included; 'No' if relevant patient groups (e.g. patients with no history of CAD) were excluded and 'Unclear' if the reported data did not allow to make a judgement.

    b. Concerns regarding applicability

Are there concerns that the included patients and setting do not match the review question? 'No' if unselected patients >18 years of age presenting to the ED with symptoms suggestive of non-ST-segment elevation myocardial infarction included; 'Yes' if the patients or the setting did not match the review question (e.g. patients with ST-segment elevation included); and 'Unclear' if unspecified.

2. Domain: Index test
    a. Risk of bias

Question 1: If a threshold was used, was it pre-specified? 'Yes' if the results for at least one pre-specified threshold were reported; 'No' if ROC-optimisation or other methods were used to define a threshold.

    b. Concerns regarding applicability

Are there concerns that the index test, its conduct, or its interpretation differ from the review question? 'No' if the assay was used according to the manufacturer's recommendations; 'Yes' otherwise.

3. Domain: Reference standard
    a. Risk of bias

Question 1: Is the reference standard likely to classify correctly the target condition? 'Yes' if the diagnosis of myocardial infarction was adjudicated independently by two qualified clinicians according to the current universal definition (Thygesen 2012) and the method included high-sensitivity troponin assay; 'No' if not (including studies using an earlier generation of troponin assay or other biomarker as a reference test for myocardial necrosis) and 'Unclear' if not reported in sufficient detail.

Question 2: Was the reference standard independent from the index test (i.e. the index test did not form part of the reference standard)? 'Yes' if different troponin assay was used; 'No' if the results from the index test were included in the reference standard; and 'Unclear' if not reported in sufficient detail.

Question 3: Were the results from the reference standard interpreted without knowledge of the results from the index test? 'Yes' or 'No' if explicitly stated, 'Unclear' if not reported.

For peer review only

b. Concerns regarding applicability

Are there concerns that the target condition as defined by the reference standard does not match the review question? 'No' if the target condition was Non-ST-segment Elevation Myocardial Infarction (NSTEMI), diagnosed according to the current universal definition; 'Yes' if patients with ST-segment elevation on the initial ECG were included or the target condition deviated in some other way from the above definition.

4. Domain: Flow and timing
   a. Risk of bias

<u>Question 1:</u> Was there an appropriate interval between the index test and reference standard? 'Yes' if the reference test was done within 24 hours of the index test; 'No' if more than 24 hours and 'Unclear' if not reported.

<u>Question 2:</u> Did all patients receive a reference standard? 'Yes' if all patients included in the analysis received a reference standard; 'No' if some patients were included in the analysis without receiving a reference standard; 'Unclear' otherwise.

<u>Question 3:</u> Did all patients receive the same reference standard? 'Yes' if all patients received the same reference standard; 'No' otherwise and 'Unclear' if insufficient information.

<u>Question 4:</u> Were all patients included in the analysis? 'Yes' if all patients enrolled in the study were accounted for and included in the analysis; 'No' if patients who were enrolled in the study were excluded from the analysis, and 'Unclear' if insufficient data were available to decide.

Rules for producing an overall risk of bias rating for each domain:

• If all signalling questions within a domain are answered 'Yes' the overall domain rating is 'Low risk of bias'

• If one or more signalling questions within a domain are answered 'No' the overall domain rating is 'High risk of bias'

• If one or more signalling questions in the domain are answered 'Unclear' while the remaining signalling questions are answered 'Yes' then the overall domain rating is 'Unclear risk of bias'.

For peer review only

Reporting checklist for protocol of a systematic review.

Based on the PRISMA-P guidelines.

Instructions to authors

Complete this checklist by entering the page numbers from your manuscript where readers will find each of the items listed below.

Your article may not currently address all the items on the checklist. Please modify your text to include the missing information. If you are certain that an item does not apply, please write "n/a" and provide a short explanation.

Upload your completed checklist as an extra file when you submit to a journal.

In your methods section, say that you used the PRISMA-P reporting guidelines, and cite them as:

Moher D, Shamseer L, Clarke M, Ghersi D, Liberati A, Petticrew M, Shekelle P, Stewart LA. Preferred Reporting Items for Systematic Review and Meta-Analysis Protocols (PRISMA-P) 2015 statement. Syst Rev. 2015;4(1):1.

		Reporting Item	Page Number
Identification	#1a	Identify the report as a protocol of a systematic review	1
Update	#1b	If the protocol is for an update of a previous systematic review, identify as such	n/a
	#2	If registered, provide the name of the registry (such as PROSPERO) and registration number	4
Contact	#3a	Provide name, institutional affiliation, e-mail address of all protocol authors; provide physical mailing address of corresponding author	1-2
Contribution	#3b	Describe contributions of protocol authors and identify the guarantor of the review	18
	#4	If the protocol represents an amendment of a previously completed or published protocol, identify as such and list changes; otherwise, state plan for documenting important protocol amendments	18

For peer review only

Sources	#5a	Indicate sources of financial or other support for the review	18-19
Sponsor	#5b	Provide name for the review funder and / or sponsor	18-19
Role of sponsor or funder	#5c	Describe roles of funder(s), sponsor(s), and / or institution(s), if any, in developing the protocol	19
Rationale	#6	Describe the rationale for the review in the context of what is already known	9-11
Objectives	#7	Provide an explicit statement of the question(s) the review will address with reference to participants, interventions, comparators, and outcomes (PICO)	11
Eligibility criteria	#8	Specify the study characteristics (such as PICO, study design, setting, time frame) and report characteristics (such as years considered, language, publication status) to be used as criteria for eligibility for the review	11-13
Information sources	#9	Describe all intended information sources (such as electronic databases, contact with study authors, trial registers or other grey literature sources) with planned dates of coverage	14
Search strategy	#10	Present draft of search strategy to be used for at least one electronic database, including planned limits, such that it could be repeated	Supplementary file
Study records - data management	#11a	Describe the mechanism(s) that will be used to manage records and data throughout the review	14
Study records - selection process	#11b	State the process that will be used for selecting studies (such as two independent reviewers) through each phase of the review (that is, screening, eligibility and inclusion in meta-analysis)	14
Study records - data collection process	#11c	Describe planned method of extracting data from reports (such as piloting forms, done independently, in duplicate), any processes for obtaining and confirming data from investigators	14-15
Data items	#12	List and define all variables for which data will be	14-15,

For peer review only

		sought (such as PICO items, funding sources), any pre-planned data assumptions and simplifications	Supplementary file
Outcomes and prioritization	#13	List and define all outcomes for which data will be sought, including prioritization of main and additional outcomes, with rationale	13
Risk of bias in individual studies	#14	Describe anticipated methods for assessing risk of bias of individual studies, including whether this will be done at the outcome or study level, or both; state how this information will be used in data synthesis	15
Data synthesis	#15a	Describe criteria under which study data will be quantitatively synthesised	16
	#15b	If data are appropriate for quantitative synthesis, describe planned summary measures, methods of handling data and methods of combining data from studies, including any planned exploration of consistency (such as I2, Kendall's τ)	16-17
	#15c	Describe any proposed additional analyses (such as sensitivity or subgroup analyses, meta-regression)	17
	#15d	If quantitative synthesis is not appropriate, describe the type of summary planned	17
Meta-bias(es)	#16	Specify any planned assessment of meta-bias(es) (such as publication bias across studies, selective reporting within studies)	15
Confidence in cumulative evidence	#17	Describe how the strength of the body of evidence will be assessed (such as GRADE)	n/a

The PRISMA-P checklist is distributed under the terms of the Creative Commons Attribution License CC-BY 4.0. This checklist can be completed online using https://www.goodreports.org/, a tool made by the EQUATOR Network in collaboration with Penelope.ai

For peer review only
